# Supplementary material for: Calcium Regulates HCC Proliferation as well as EGFR Recycling/Degradation and Could Be a New Therapeutic Target in HCC
Source: Cancers (Basel). 2019 Oct 18;11(10):1588. doi: 10.3390/cancers11101588 (PMC6826902; doi:10.3390/cancers11101588)
Supplement: Supplementary file 1 [file cancers-11-01588-s001.pdf]

Supplementary Materials

Calcium Regulates HCC Proliferation as well as EGFR Recycling/Degradation and Could Be a New Therapeutic Target in HCC

Teresa Maria Elisa Modica, Francesco Dituri, Serena Mancarella, Claudio Pisano, Isabel Fabregat and Gianluigi Giannelli

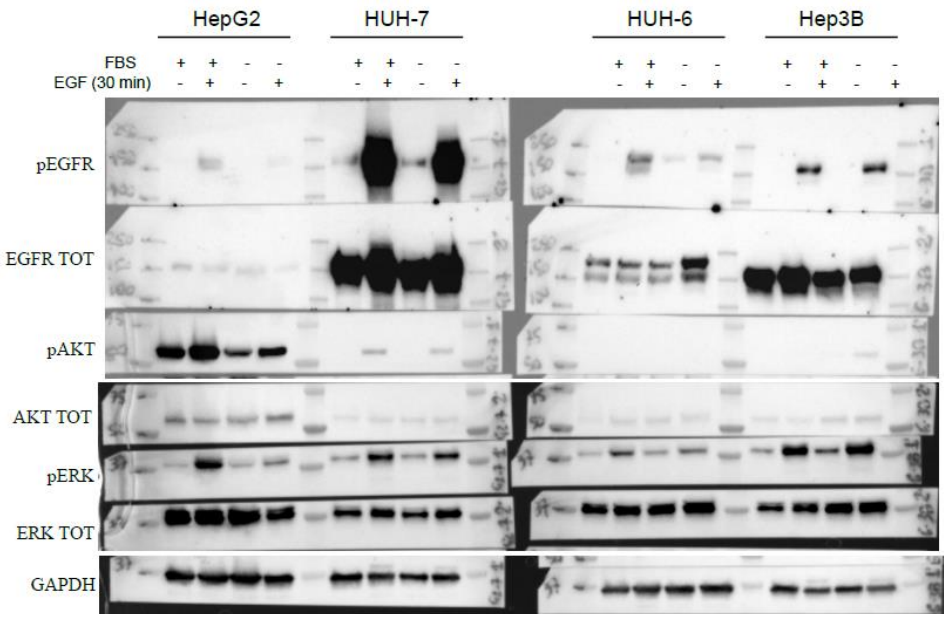

Figure S1. Western blot analysis of EGFR pathway activation in HepG2, HUH-7, HUH-6, and Hep3B cell lines.

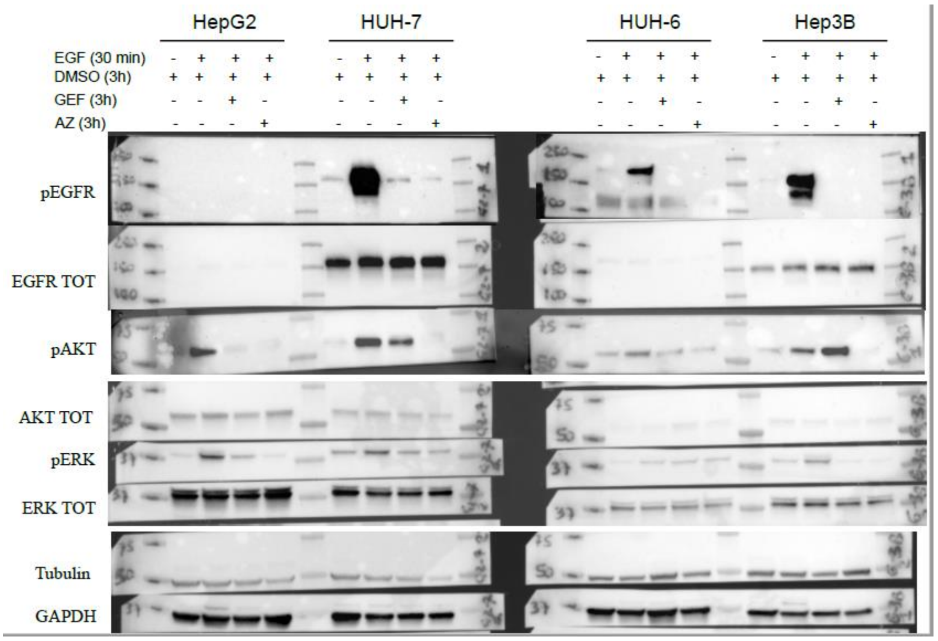

Figure S2. Western blot analysis of HepG2, HUH-7, HUH-6, and Hep3B starved cell lines treated with GEF IC50 or AZ IC50 (as indicated in Table 1) (DMSO as control) for 3 h before stimulation with 100 ng/mL of EGF for 30 min.

A.

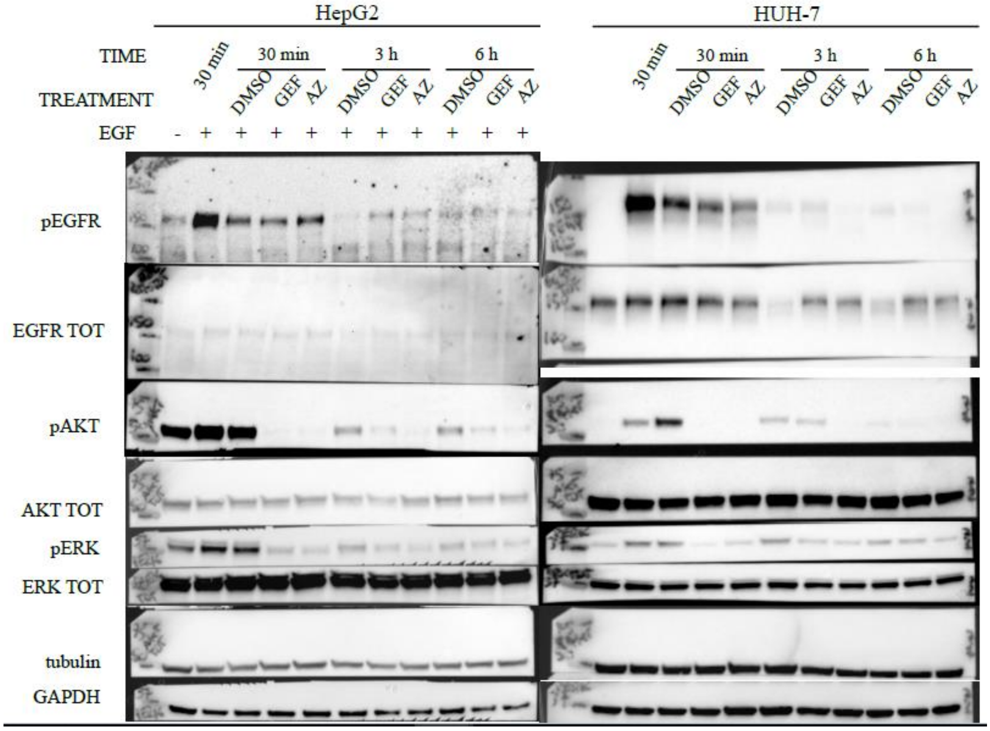

B.

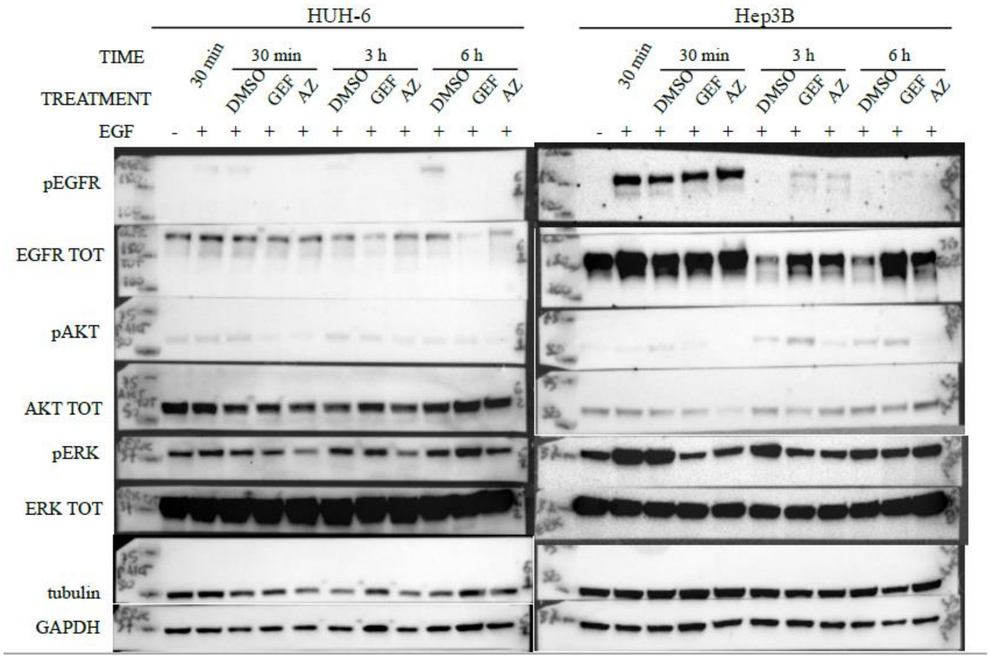

Figure S3. A and **B**: Western blot panels of HepG2, HUH-7, HUH-6, and Hep3B starved cell lines stimulated with 100 ng/mL of EGF for 30 min before and during treatment with GEF or AZ IC50 (as indicated in Table 1) (DMSO as control). Treatments were performed for 30 min, 3 h and 6 h.

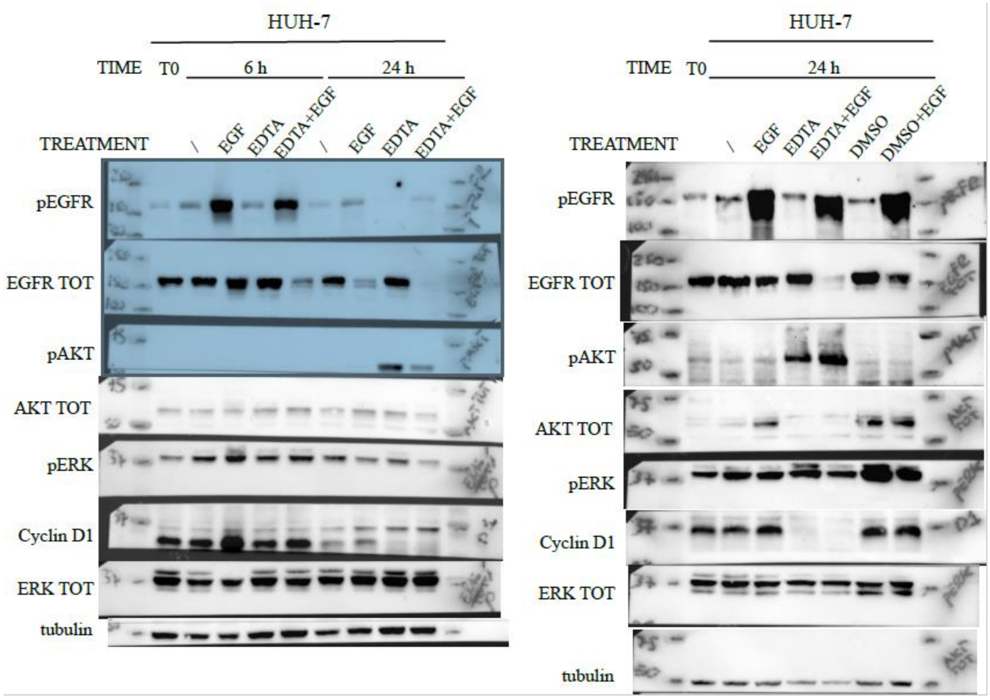

Figure S4. Starved HUH-7 cells (T0) were left untreated (/) (0% FBS as CTR) or treated with 100 ng/mL EGF, 2 mM EDTA, 0.5% DMSO, or combined compounds (as indicated in the figures). The cell signaling cascade was analyzed by western blot after 6 h and 24 h.

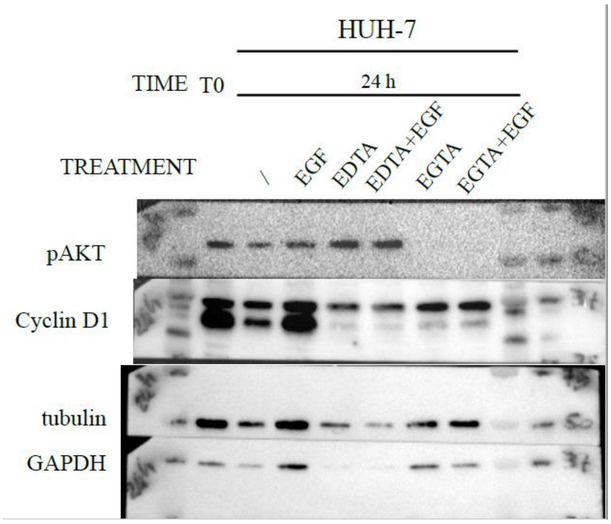

Figure S5. HUH-7 cells treated with EDTA or EGTA for 24 h with or without EGF were analyzed by western blot.

A.

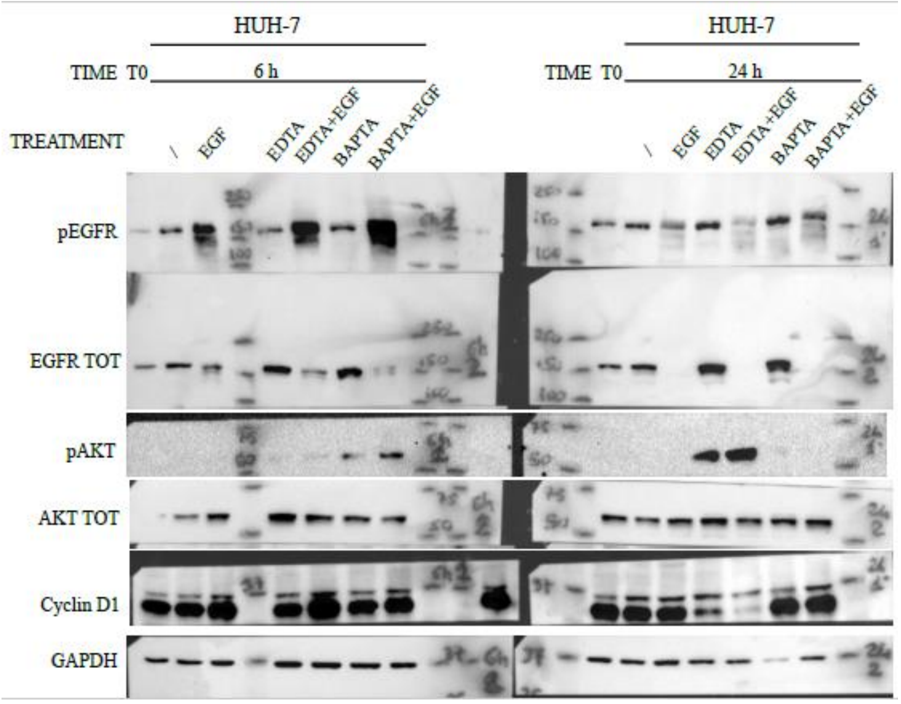

B.

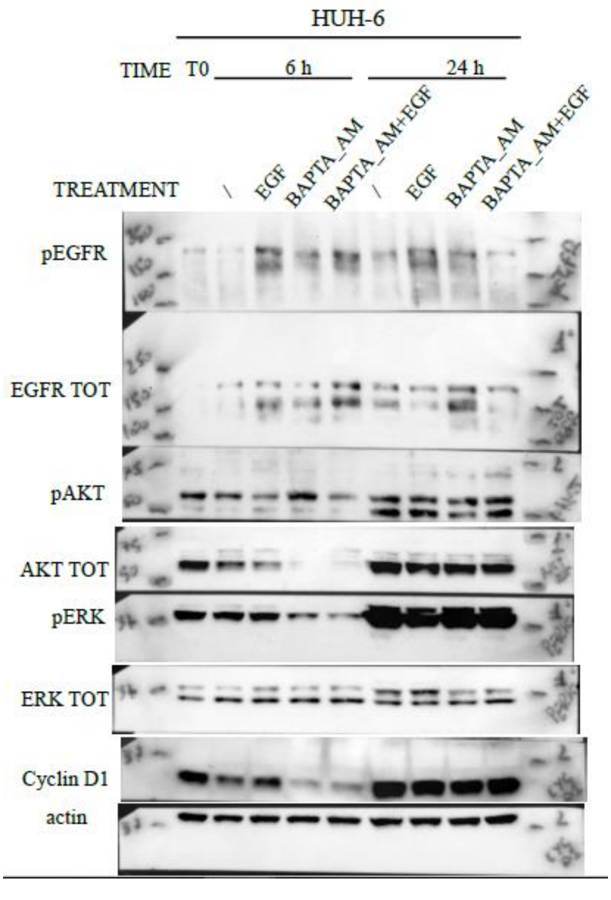

Figure S6. A and **B**: Starved HUH-7 and HUH-6 cells (T0) were left untreated (as CTR) or treated with 2 mM EDTA or 10 μM BAPTA_AM with or without 100 ng/mL EGF. The cell signaling cascade was analyzed by western blot after 6 h and 24 h.

A.

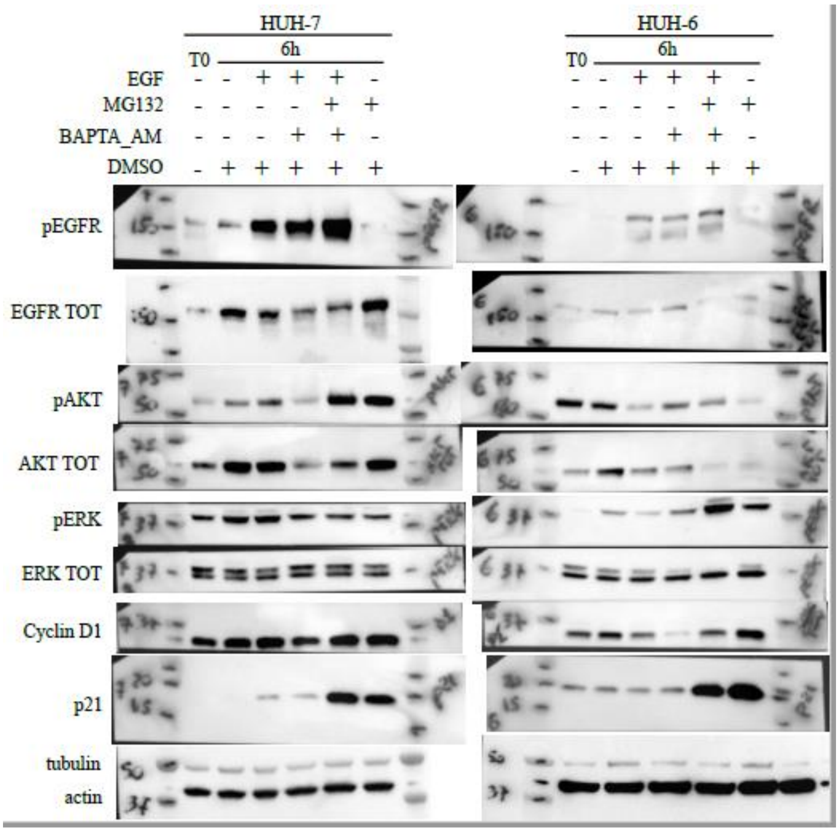

B.

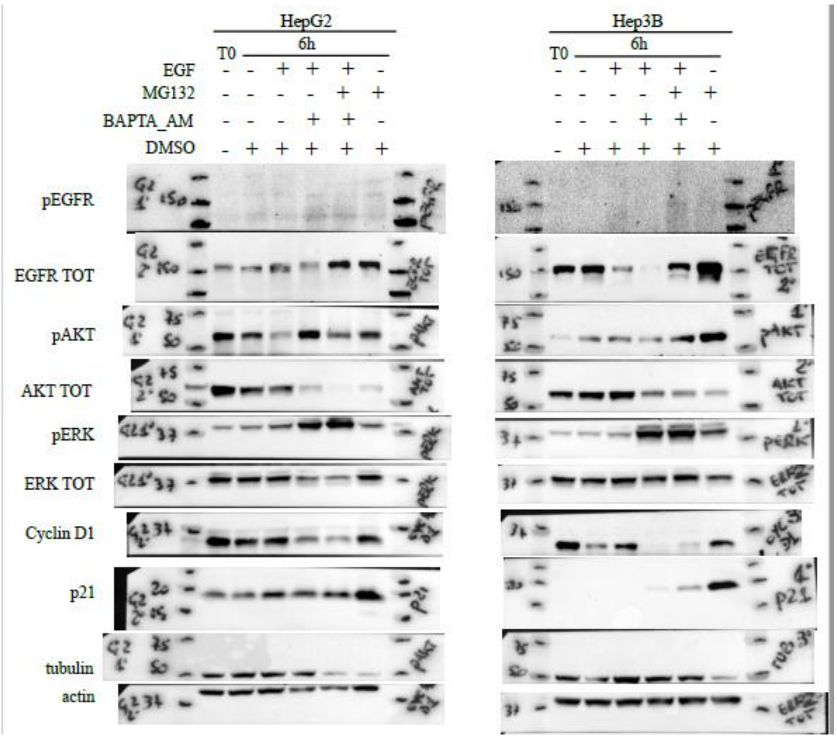

Figure S7. A and **B**: Starved HUH-7, HUH-6, HepG2, and Hep3B cells (T0) were left untreated (as CTR) or treated with 10 μM BAPTA_AM. After 30 min, 40 μM MG132 were added for a further 30 min. 100 ng/mL EGF were added for a total time of 6 h before cells harvesting.

© 2019 by the authors. Licensee MDPI, Basel, Switzerland. This article is an open access article distributed under the terms and conditions of the Creative Commons Attribution (CC BY) license (http://creativecommons.org/licenses/by/4.0/).
